# Shaping microbiology for 75 years: highlights of research published in *Microbiology*. Part 1 - Physiology and growth

**DOI:** 10.1099/mic.0.001356

**Published:** 2023-06-28

**Authors:** Michael Brockhurst, Jennifer Cavet, Stephen P. Diggle, David Grainger, Riccardo Mangenelli, Hana Sychrova, Isabel Martin-Verstraete, Martin Welch, Tracy Palmer, Gavin H. Thomas

**Affiliations:** ^1^​ Division of Evolution, Infection and Genomics, University of Manchester, Michael Smith Building, Dover Street, Manchester M13 9PT, UK; ^2^​ Lydia Becker Institute of Immunology and Inflammation, School of Biological Sciences, Faculty of Biology Medicine and Health, University of Manchester, Manchester M13 9PT, UK; ^3^​ Center for Microbial Dynamics and Infection, School of Biological Sciences, Georgia Institute of Technology, Atlanta, GA, USA; ^4^​ School of Biosciences, University of Birmingham, Edgbaston, Birmingham B15 2TT, UK; ^5^​ Department of Molecular Medicine, University of Padova, 35121 Padova, Italy; ^6^​ Institute of Physiology of the Czech Academy of Sciences, Laboratory of Membrane Transport, 14200 Prague 4, Czech Republic; ^7^​ Institut Universitaire de France, Paris, France; ^8^​ Department of Biochemistry, University of Cambridge, Cambridge, UK; ^9^​ Microbes in Health and Disease Theme, Newcastle University Biosciences Institute, Newcastle University, Newcastle upon Tyne, NE2 4HH, UK; ^10^​ Department of Biology, University of York, Wentworth Way, York, UK

## Introduction

The *Journal of General Microbiology* was first published in 1947, two years after the founding of the Society for General Microbiology. Although both names have changed, to *Microbiology* and the Microbiology Society, respectively, the former names capture a key aspect of the journal, in that it is inclusive of all microbes, from viruses through to fungi, protozoa and algae. Except for animal and plant virology, which branched off in 1967 to create the *Journal of General Virology*, this ambition continues to this day. The word ‘general’ was a counterpoint to the many specific applied microbiology journals that existed in this period, in the diary and brewing industries, for example. Whilst the Society was founded to bring together diverse groups of researchers, doing more fundamental research, the journal encompasses both fundamental and applied research, which can be seen from articles published right from its first issue to the current day.

In this two-part celebratory article, the team of senior editors associated with the journal at the start of our anniversary year have reviewed highly cited and influential papers published in the journal's 75 year history. The 40 or so selected papers, which date from 1956 to 2014, highlight some favourite themes, under a number of broader topic areas. While not comprehensive, we hope it will highlight of areas where the journal has championed important work and will encourage continued submission of strong research as the journal as it continues beyond its 75th year.

## Understanding microbial growth and physiology

The ability to culture microbes in the laboratory in controlled and reproducible ways was integral to the work of the early pioneers of our subject. This enabled experiments to tease apart how they resource food, convert this to energy and then build new biomass. We start with a series of early fundamental papers, focused on microbial physiology and growth.

Our earliest highlighted paper from 1956 is a classic on the use of continuous culture for the growth of bacteria, from scientists at the Microbiological Research Department at Porton Down, Wiltshire, an important research centre for microbiology in the UK from this period [1]. The paper, by Herbert, Elsworth and Telling, provides some of the first quantitative data comparing batch and continuous culture methods in the context of theoretical models of growth. At the time the bacterium *Aerobacter cloacae* (now *Enterobacter cloacae *, a fast-growing non-pathogen that can grow on defined media) was popular for such studies. This work was followed shortly by two other important papers. The first, from Schaechter and colleagues in 1958, determined the influence of media composition and temperature on cell size [2]. The second, from Contois in 1959, provided a mathematical model for bacterial growth in continuous culture [3]. The classic paper of Bauchop and Elsden followed these in 1960, examining the amount of biomass produced by *Enterococcus faecalis* when grown on glucose as the sole energy source and only using substrate-level phosphorylation for energy production. Using this system, they established a direct relationship between the amount of energy source and biomass. Knowing how ATP was generated from glucose they derived a measure for growth yield, termed *Y*_ATP_ [4]. This associated calculation gave microbiologists a tool to interrogate the bioenergetics of other microbes in a quantitative way and reveal differences between them. Ultimately this underpinned the discovery of the diverse fermentation pathways used by bacteria. Together, these papers were important in creating a solid theoretical and experimental framework for studying microbial growth. In a much more recent review by Hoskisson and Gibson [5], the authors describe modern applications of chemostats and quantitative growth measurements for both fundamental and biotechnological applications and these papers will continue to be cited for generations to come.

Yeast physiology and metabolism also featured in early classic papers. De Deken in 1966 elucidated the generality of a process called the ‘Crabtree effect’, which is when yeast growing on certain sugars in the presence of oxygen use fermentative metabolism rather aerobic respiration. De Deken determined that this was due to a regulatory system that resulted in repression of the respiratory system when growing on selected sugars, and that this occurred in many yeasts in addition to the model brewer’s yeast *Saccharomyces cerevisiae* [6]. More recently, an important paper looked at the physiology of *S. cerevisiae* during anaerobic glucose-limited culture, another growth mode that is available to this metabolically flexible class of microbes [7]. This uncovered important new insights into how acetate, as a metabolic end product, elicits an uncoupling effect and inhibits cell growth.

This transition between aerobic and anaerobic growth has also been the topic of many other papers in the journal. Here *Escherichia coli* has often served as the model system, as being a facultative anaerobe it can grow easily in the laboratory either with or without oxygen and can quickly transition between these modes. An elegant paper in the early days of molecular genetics described a mutant of *E. coli* that cannot grow in anaerobic conditions using fumarate as the sole electron acceptor for respiration [8]. The mutant, isolated by Lambden and Guest, turned out to be in the gene encoding a key transcriptional regulator called FNR, which is now known to use a labile [4Fe–4S] cluster to directly sense the presence of oxygen and either activate or repress expression of genes involved in the aerobic/anaerobic transition. While this paper is the citation classic, 3 years earlier, Cole and Ward described mutants with decreased anaerobic growth on nitrite that mapped to a gene they called *nirA*. This was later found to be identical to *fnr* [9], so the journal can rightly claim to have published the discovery of *fnr* twice!

As well as the regulatory mechanisms involved in this aerobic–anaerobic transition, the biochemical and physiological changes in the presence and absence of oxygen also highlight the use of metals in enzymes, where the journal has also published important papers. The use of metals in respiratory enzymes, but also more widely in many other enzymes, has prompted important reviews by Hughes and Poole [10] and Gadd [11]. In the former, the authors hoped to trigger a field of ‘inorganic microbiology’, where microbiologists would consider not only which metals are required for growth, but also in which chemical form they are found. This can have important outcomes *in vivo*, as metals that are essential at low concentrations can be toxic at higher concentrations, and bacteria have evolved many ways to control the intracellular levels of potentially toxic metals such as copper, for example using the CopAB system in the plant pathogen *Xanthomonas axonopodis* [12]. Iron needs to be acquired at higher levels than many other metals due to its function in numerous proteins and enzymes, and bacteria have elegant ways to scavenge this from the environment. The discovery of how the pathogen *Pseudomonas aeruginosa* acquires iron from organic sources was described in an important paper from 2000, where an elegant genetic study revealed two distinct uptake routes for iron capture via haem [13], both of which are regulated through the iron-responsive Fur transcription factor.

One consequence of high concentrations of metals such as iron is the generation of reactive oxygen species or ROS from molecular oxygen (O_2_). The appreciation that ROS are generated constantly during aerobic growth, through electron leakage from respiratory enzymes and by host organisms to kill invading microbes, triggered many studies from the mid-1990s onwards. Particularly strong contributions to *Microbiology* have shed light on adaptive strategies that allow obligate anaerobes to tolerate O_2_ and to scavenge ROS. Anaerobes are ubiquitous and include bacteria of environmental, medical or biotechnological interest, as well as commensals and pathogens of the gut microbiota. Consequently, they have been well studied by microbiologists from the outset of both the Society and the journal.

Most anaerobes, despite their name, can tolerate transient exposure to air, low-O_2_ tensions or ROS, and many of the environments inhabited by anaerobes pose these stresses from time to time. An important early paper by O’Brian and Morris in 1971 examined how the soil anaerobe *Clostridium acetobutylicum* can survive in the presence of low-O_2_ tensions or tolerate high-O_2_ tensions with an arrest of growth, which can resume when cells return to anaerobiosis [14]. A similar process of transient tolerance also occurs in other Clostridia and Bacteroidetes and in the anaerobic sulfate-reducing bacterium *Desulfovibrio desulfuricans* [15]. The O_2_ toxicity can be direct or indirect, the latter through the formation of ROS. Both oxygen and ROS can poison key enzymes involved in anaerobic growth, a recent example of this being published in the journal [16], and many anaerobes possess enzymes that allow rapid removal of oxygen as the mechanism of protection. A membrane-bound quinol O_2_ reductase and an atypical cytochrome c oxidase can use H_2_ to reduce O_2_ in *Desulfovibrio vulgaris* for this purpose [17, 18]. In another solution to a similar problem, *C. acetobutylicum* uses novel non-haem iron proteins called reverse rubrerythrins as part of an NAD(P)H-dependent O_2_ reductase to protect against oxidative stress [19], which was also recently confirmed in the major enteropathogen *Clostridioides difficile* [20].

As well as dealing with O_2_ directly, microbes have diverse ways to handle ROS in complex adaptive responses. While *Clostridium perfringens* has surprisingly classical (in non-obligate anaerobes) enzymes of ROS detoxification, such as superoxide dismutase, catalase and hydroxyperoxide reductase [21], other anaerobes use alternative enzymes like superoxide reductases and peroxide reductases, thereby avoiding the production of O_2_ as a by-product. These mechanisms are not restricted to anaerobic bacteria either, and the role of a copper- and zinc-containing superoxide dismutase in the pathogenic fungus *Candida albicans* was established in a paper published in the journal [22]. An apparently novel mechanism discovered in *C. difficile* confers a high level of resistance against peroxide via the secretion of a glutamate dehydrogenase-like enzyme through an unknown mechanism [23] and confers protection in a mouse model of infection. Clearly there is still lots to learn about how microbes are able to consume O_2_, eliminate endogenously produced ROS in aerated cultures, adapt their metabolism and efficiently repair damaged molecules.

## Looking into cells and their behaviour

The interesting behaviours in which microbes engage often involve processes at the cell envelope or within organelles, where microscopy with direct visualization of microbial cells has been a key experimental tool. An important paper published in the journal was the first optimization of green fluorescent protein (yEGFP) for use in yeast [24], initially for the pathogenic yeast *C. albicans*. This tool was used mainly as a reporter of gene expression, although it later became invaluable for protein localization and/or morphology studies in many yeast species.

One fascinating cellular process, well studied in many different microbes, is chemotaxis, the understanding of which has led to in-depth understanding of sensing and signal transduction pathways. A significant early methodological paper was published in the journal in 1973 by Julius Adler, considered by many to be the pioneer in this field [25]. Adler and colleagues had already refined Pfeffer’s original ‘capillary accumulation assay’ (developed in the 1880s) for investigating bacterial motility and chemotaxis, and had begun to apply it to the quantitative study of these behaviours in *E. coli *. In essence, a capillary is loaded with a defined buffer containing an attractant chemostimulus. One end of the capillary is then placed into a suspension of *E. coli* cells. As the attractant diffuses out of the capillary, a gradient is generated and the bacteria move up that gradient and into the capillary. After a predetermined amount of time, the capillary contents (cell counts, measured as colony-forming units) are assayed. This simple assay allowed Adler to define the best conditions for assaying bacterial chemotaxis and set the scene for almost all subsequent work in the field and consequently has been (and continues to be) cited many times.

As a Gram-negative organism, *E. coli* has a complex cell envelope comprising both an outer and inner membrane. One controversial paper, which was discounted for many years by the community, was published by Bayer in 1968 [26]. In this work, plasmolysed cells of *E. coli* were examined by transmission electron microscopy. Plasmolysis occurs when bacteria are placed in hypo-osmotic medium, with shrinkage of the cytoplasm leading to separation of the cytoplasmic membrane from the cell wall and outer membrane. The micrographs revealed areas where, unexpectedly, the two membranes remained in contact despite cytoplasmic shrinkage. Bayer and others proposed that these contact sites functioned in the export of lipopolysaccharides. These sites of membrane contact became known as ‘Bayer junctions’ or ‘Bayer patches’, although for many years they were dismissed as an artefact arising from the chemical fixation process used to prepare the cells for electron microscopy. Recently structural and biochemical analysis of the lipopolysaccharide transport machinery (LPT) have shown that it forms a transenvelope machine that connects both membranes. Since the structure formed during this process is resistant to plasmolysis, it likely gives rise to the contact sites seen in Bayer’s original images.

While we considered growth of bacteria in liquid culture in the previous section, microbes can also be grown on surfaces as colonies, the kinetic study of which was described by Pirt in a classic paper in 1967 [27]. Much more recently, the study of growth of bacteria on solid surfaces has been used to dissect the biofilm phenotype, in species such as *Bacillus subtilis* and *P. aeruginosa *. The formation of biofilms requires some matrix or ‘glue molecules’ to be secreted to hold the structure together, which is often a secreted polysaccharide. However, an important paper in the journal discovered that in the pathogen *Acinetobacter baumannii *, there is an essential role for the cell surface pili in this process and that biofilm development is abolished when biosynthesis of the pili, via a novel chaperone-usher system, is disrupted [28].

The implications of life in biofilms was the subject of another classic paper from Jan-Ulrich Kreft in 2004, where he used mathematical modelling to argue that bacteria within biofilms are behaving altruistically [29]. Unlike in highly agitated chemostat cultures, where bacteria divide as quickly as possible to keep the population going, the spatial structure inherent to microbial biofilms that form in microcolonies is, for example, necessary for the bacteria within this to grow more slowly in a cooperative community to ultimately reach a high growth yield. The hypothesis provides insight into the evolution of altruism and offers a uniquely microbial solution to the longstanding ‘tragedy of the commons’ social dilemma by limiting the invasion of non-cooperating cheats into the stable microcolonies.

In part 2 we will continue our journey and look a papers based around the broader themes of microbial communities and evolution as we complete our journey through 75 years of the journal’s most impactful papers.
